# Optimal sedation/anesthesia practices for painless gastroscopy in older adults: a randomized trial

**DOI:** 10.3389/fmed.2025.1554937

**Published:** 2025-05-22

**Authors:** Jing-Zhen Liu, Xin Liu, Duan-Gao Wei

**Affiliations:** ^1^Department of Emergency, The Third Affiliated Hospital of Nanjing Medical University, The Second People’s Hospital of Changzhou, Changzhou, Jiangsu, China; ^2^Department of Anesthesiology, Northern Jiangsu People’s Hospital, Northern Jiangsu People’s Hospital Affiliated to Yangzhou University, Yangzhou, Jiangsu, China

**Keywords:** depth of sedation, gastroscopy, aged adults, sedation, painless

## Abstract

**Objective:**

The aim of this study is to conduct a preliminary exploration of the optimal sedation depth for painless gastroscopy in older adults.

**Methods:**

Sixty older adults who underwent painless gastroscopy in April 2023 at Northern Jiangsu People’s Hospital, Jiangsu Province, were included in the study and randomly assigned to four groups: minimal sedation (Group I), moderate sedation (Group II), deep sedation (Group III), and anesthesia (Group IV). Data were collected on sedation depth after induction and titration (T1), swallowing response during gastroscope insertion (T2), and bucking during the procedure (T3).

**Results:**

Following T1, both systolic blood pressure (SBP) and diastolic blood pressure (DBP) showed a significant decrease, with change rates being notably higher in groups III and IV compared to groups I and II (*P* < 0.05). The failure rate of gastroscope insertion was significantly higher in groups I and II than in groups III and IV (*P* < 0.05). At T2, bucking and body movement scores were substantially lower in groups III and IV than in groups I and II (*P* < 0.05). After T2, the heart rate (HR) change rates in groups III and IV were significantly lower than in groups I and II (*P* < 0.05). Additionally, SBP and DBP were further reduced, with change rates being markedly higher in groups III and IV compared to groups I and II (*P* < 0.05). At T3, the incidence of bucking and body movements was significantly lower in groups II, III, and IV than in group I (*P* < 0.05).

**Conclusion:**

Deep sedation and anesthesia are more suitable for older adults undergoing painless gastroscopy in terms of sedation depth.

**Clinical trial registration:**

https://www.chictr.org.cn/showproj.html?proj=191819, ChiCTR2300069999.

## 1 Introduction

The appropriate depth of sedation or anesthesia is closely linked to patient comfort, anesthetic effectiveness, patient satisfaction, and medical safety during painless gastroscopy, particularly in older adults with poor baseline health, multiple comorbidities, and high safety requirements. Although there is substantial practical experience with anesthesia for painless gastrointestinal endoscopy, rigorous, targeted scientific research on the optimal depth of sedation or anesthesia for older adults remains limited. Over the past decade, expert consensus guidelines on painless gastrointestinal endoscopy in China have emphasized the importance of selecting the appropriate sedation or anesthesia depth (1–4). Similarly, international guidelines recommend the gradual titration of sedative or anesthetic medications to achieve the desired depth (5–7). However, the precise definition of the appropriate sedation or anesthesia depth remains a topic of debate. Influenced by international practices, some scholars tend to prefer moderate or even minimal sedation for painless gastrointestinal endoscopy, while others advocate for deeper sedation or even general anesthesia (8–13). In this context, this randomized controlled trial aimed to preliminarily explore the optimal depth of sedation or anesthesia for painless gastroscopy, a common diagnostic and therapeutic procedure, in older adults.

## 2 Materials and methods

### 2.1 General information

The study received approval from the Medical Ethics Committee of Northern Jiangsu People’s Hospital, Jiangsu Province (Approval No. 2023ky061), and all participants provided informed consent. Additionally, the study was registered with the Chinese Clinical Trial Registry (Registration website: https://www.chictr.org.cn/showproj.html?proj=191819, Registration number: ChiCTR2300069999, Registration date: 30/3/2023). A total of 60 older adults who underwent painless gastroscopy at the Endoscopy Center of Northern Jiangsu People’s Hospital, Changzhou, China, from April 10th to April 25th, 2023, were included in this study. Inclusion criteria were as follows: age ≥ 60 years; body mass index (BMI) between 18 and 30 kg/m^2^; American Society of Anesthesiologists physical status classification < grade III; general gastroscopy; ability to communicate effectively, normal hearing, and clear responses; informed consent and voluntary participation. Exclusion criteria were: patients scheduled for other endoscopic procedures or surgeries, such as foreign body removal or gastrointestinal polypectomy; and patients with contraindications or relative contraindications to outpatient anesthesia. The majority of the research team members are anesthesiologists. Both the study design and its implementation were carried out by anesthesiologists.

### 2.2 Methods

#### 2.2.1 Gastroscopy operations

Gastroscopy was conducted by a qualified gastrointestinal endoscopist with a minimum of 5 years of specialized experience. Participants were instructed to slowly swallow 0.1 g of dyclonine hydrochloride mucilage 10-15 min before the procedure. The endoscopist proceeded with inserting the scope only after the target depth of sedation or anesthesia was reached through induction in each group.

#### 2.2.2 Randomization, concealment and blinding

Participants were randomly assigned to one of four groups—minimal sedation (Group I), moderate sedation (Group II), deep sedation (Group III), and anesthesia (Group IV)—with 15 participants in each group, using the random number table method. Each group received different levels of sedation or anesthesia and varied titration regimens. The random number table and grouping criteria were established by an independent individual, and both investigators and participants were blinded to the group assignments of subsequent participants. Due to the different sedation and anesthesia depths, blinding of participants and healthcare personnel was not feasible. Nonetheless, the purpose and primary evaluation criteria of the study were not disclosed to the anesthesiologists, endoscopists, and endoscopy nurses during the procedure. Data compilation and analysis were carried out by a separate evaluator.

#### 2.2.3 Titration methods and sedation failure and remedies for target depth of sedation/anesthesia

Participants in each group were sedated with propofol and underwent anesthesia induction according to the doses specified in Table 1. If the target depth of sedation or anesthesia was not achieved, additional doses were administered according to the group’s specific titration regimen, with at least a 1-min interval between doses. If sedation or anesthesia depth exceeded the target after induction or additional doses, the participant was considered a failure and withdrawn from the study. In cases of sedation failure, the procedure was paused for treatment, or a deeper level of sedation or anesthesia was implemented if the endoscopist could not insert the scope within two attempts at the target depth or if severe bucking or body movements occurred. When sedation failure was identified, the anesthesiologist could administer remedial sedation, such as additional propofol, to complete the gastroscopy. If blood pressure (BP) dropped below 70% of the baseline value or systolic BP (SBP) fell below 90 mmHg (1 mmHg = 0.133 kPa), participants were given an intravenous injection of 8-10 μg of norepinephrine. For a heart rate (HR) below 60 beats per minute, participants received 0.5 mg of atropine intravenously. If oxygen saturation (SpO_2_) fell below 90%, the airway was managed by tilting the head back and lifting the lower jaw, or applying gentle pressure to the mandibular angle to enhance respiratory amplitude.

**TABLE 1:** Anesthesia induction and titration doses of propofol were administered in the different groups.

Groups	Anesthesia induction dose (mg⋅kg^–1^)	Additional induction dose (mg⋅kg^–1^⋅time^–1^)
Group I	0.5	0.2
Group II	1.0	0.2
Group III	1.5	0.5
Group IV	2.5	0.5

Group I, Minimal sedation; Group II, Moderate sedation; Group III, Deep sedation; Group IV, Anesthesia.

#### 2.2.4 Main evaluation indicators

The main evaluation indicators included the depth of sedation or anesthesia at the end of induction and titration (T1), vital signs measured before and after T1, frequency of swallowing, bucking scores, body movement scores, and the number of gastroscope insertions required during the procedure (T2). Additionally, vital signs were recorded before and after T2 and bucking and body movement scores were assessed during gastroscopy (T3).

#### 2.2.5 Evaluation criteria

The depth of sedation or anesthesia was assessed using the Ramsay Sedation Scale:(1) scores of 2-3 indicated minimal sedation; a score of 4 indicated moderate sedation; scores of 5-6 indicated deep sedation; and scores greater than 6 with no response to painful stimuli indicated anesthesia. Bucking was evaluated using a four-level scale:(14) Grade 0, no bucking; Grade 1, mild bucking that could be alleviated by changing the patient’s head position or adjusting the gastroscope, without affecting the procedure; Grade 2, moderate bucking requiring suspension of the procedure and deepening of anesthesia; and Grade 3, severe bucking accompanied by shortness of breath and decreased SpO_2_, necessitating scope withdrawal, positive pressure ventilation via mask, and deepening of anesthesia. Body movement scores were categorized into four grades:(14) Grade 0, no body movements; Grade 1, slight involuntary limb movements that did not impact the endoscopy and did not require additional propofol; Grade 2, hand grasping of the gastroscope by the patient, affecting the endoscopist’s examination and necessitating additional anesthetic drugs; and Grade 3, restlessness and complete non-cooperation, requiring termination of the examination, scope withdrawal, and deepening of anesthesia.

#### 2.2.6 Quality control

Quality control measures included: (1) enhancing the organization of the research team and clarifying processes and rules; (2) appointing an independent inspector; and (3) having a third party handle data registration and statistical analysis.

#### 2.2.7 Sample size calculation

Based on our pre-experiment results, the sedation success rate was the primary evaluation indicator, with a maximum rate of 90% and a minimum rate of 27%. During our preliminary study, we conducted sedation trials on a total of 15 patients, 8 under mild sedation and 7 under moderate sedation. The primary outcome measure was the sedation success rate. Among these 15 patients, only 4 achieved successful sedation, resulting in a success rate of just 27%. Based on our clinical experience, deep sedation and general anesthesia can almost always achieve successful sedation, close to a 100% success rate. Therefore, we decided to use 90% as the assumed maximum sedation success rate for our calculations.

Using these parameters, we calculated the required sample size to ensure that the study could detect significant differences between the groups. The effect size (Cohen’s h) was assumed to reflect a medium-sized difference between the sedation success rates of the groups. Based on the pre-experiment data, the highest success rate was 90% (*P*1 = 0.9) and the lowest was 27% (*P*2 = 0.27). Cohen’s h was calculated using the formula: $h=2.\text{arcsin}(\sqrt{p\,1}-2.\text{arcsin}(\sqrt{p\,2}$

Substituting the values, we found that Cohen’s h was approximately 1.36. This effect size indicates a significant difference in sedation success rates, which is clinically meaningful. The sample size was then calculated using GPower software. The parameters were set as follows: significance level (α) of 0.05, power (1-β) of 0.80, effect size (Cohen’s h) of 1.36, and 4 groups. A Chi-square test was used for comparison of proportions. According to the GPower software calculation, the required sample size per group was approximately 10 participants. However, to account for potential dropouts and missing data, we decided to recruit 15 participants per group, resulting in a total sample size of 60 participants.

#### 2.2.8 Statistical analysis

20.0 software was used for data analysis. Normality was tested using the Shapiro-Wilk method, and *P* > 0.10 was considered to conform to a normal distribution. The measurement data with normal distribution and homogeneity of variance were expressed as mean ± standard deviation (± s). One-way ANOVA was used for comparison between groups, and LSD method was used for pairwise comparison if there were significant differences. Measurement data with abnormal distribution or uneven variance were expressed as median and interquartile range [M (P25, P75)]. The Kruskal-Wallis test was used for multiple group comparisons, followed by Dunn-Bonferroni correction for pairwise comparison when necessary. Count data were expressed as frequency, and Chi-square test was used (Fisher’s exact test was used if theoretical frequency was less than 5). All tests were two-sided, and *P*-values of less than 0.05 were considered statistically significant. For multiple comparisons, *P*-values were adjusted with the use of the Bonferroni procedure to control type I error.

## 3 Results

### 3.1 Comparison of general information

This study included 60 participants, with 15 participants per group. All patients were American Society of Anesthesiologists physical status classification II. There were no statistical differences in general characteristics, such as gender, age, and BMI (*P* > 0.05) (Table 2).

**TABLE 2:** Demographic and clinical characteristics of participants stratified by intervention groups.

Characteristics	Group I (*n* = 15)	Group II (*n* = 15)	Group III (*n* = 15)	Group IV (*n* = 15)	Statistic	*P*
Gender [n (%)]						
- Male	9 (60.0)	8 (53.3)	8 (53.3)	9 (60.0)	0.271	0.965
- Female	6 (40.0)	7 (46.7)	7 (46.7)	6 (40.0)		
Age (years)	71.1 ± 7.0	69.9 ± 5.5	71.9 ± 7.2	69.8 ± 9.3	0.279	0.840
BMI (kg/m^2^)	23.4 ± 2.2	24.7 ± 3.2	23.5 ± 1.9	24.4 ± 3.0	0.913	0.441
Hypertension [n (%)]	13 (86.7)	13 (86.7)	12 (80.0)	14 (93.3)	1.333	0.721
Diabetes [n (%)]	10 (66.7)	11 (73.3)	9 (60.0)	12 (80.0)	1.875	0.599
Coronary heart disease [n (%)]	2 (13.3)	1 (6.70)	2 (13.3)	2 (13.3)	–	>0.999

Group I, Minimal sedation; Group II, Moderate sedation; Group III, Deep sedation; Group IV, Anesthesia. Quantitative data, mean ± SD; Qualitative data: n (%). BMI, body mass index. Statistical tests, One-way ANOVA for continuous variables; Chi-square for categorical variables.

### 3.2 Change rates of vital signs after anesthesia induction and titration (T1)

The baseline HR, SBP, diastolic blood pressure (DBP), and SpO_2_ were not significantly different among these groups (*P* > 0.05). After T1, the changes in HR and SpO_2_ were not statistically significant among the four groups (*P* > 0.05). However, the changes in SBP and DBP were statistically significant (*P* < 0.05) (Table 3).

**TABLE 3:** Changes in vital signs before and after anesthesia induction and titration (T1) in the different groups.

Characteristics [%, IQR]	Group I (*n* = 15)	Group II (*n* = 15)	Group III (*n* = 15)	Group IV (*n* = 15)	*P*
HR change rates after induction	0 (–1.601,0)	–7.827 (–11.504, –1.27)	–3 (–11.765,7.937)	1.471 (–3.615,6.944)	0.216
SBP change rates after induction	–1 (–5.211,1.25)	–0.925 (–7.528,14.474)	–17.83 (–27.103, –11.475)*^#^	–16.279 (–25.197, –9.167)*	0.001
DBP change rates after induction	–6.742 (–10.518,1.667)	–7.651 (–9.238,1.897)	–11.842 (–19.697, –6.154)	–11.94 (–16, –4.478)^#^	0.06
SpO_2_ change rates after induction	0 (0,0.505)	0 (0,1.01)	0 (0,0)	0 (–4.04,0)	0.017

Group I, Minimal sedation; Group II, Moderate sedation; Group III, Deep sedation; Group IV, Anesthesia.

*P < 0.05 compared to Group I;

^#^*P* < 0.05 compared to Group II. Multiple comparisons were adjusted using the Bonferroni method. Data reporting methods, median and interquartile range [M (P25, P75)]. HR, Heart Rate; SBP, Systolic Blood Pressure; DBP, Diastolic Blood Pressure; SpO_2_, Peripheral Oxygen Saturation. Statistical tests, the Kruskal-Wallis test was used for comparison between groups, and Dunn-Bonferroni correction was performed for pairwise comparison.

In all participants who experienced significant decreases in SBP and DBP, intravenous administration of norepinephrine (8-10 μg) restored blood pressure to normal levels without causing any subsequent adverse events.

### 3.3 Situation at gastroscope insertion (T2)

#### 3.3.1 Failure rate of gastroscope insertion

Significant differences were observed in the failure rate of gastroscope insertion among the four groups (*P* < 0.05). Groups I and II had a notably higher failure rate compared to groups III and IV (*P* < 0.05) (Table 4).

**TABLE 4:** Failure rates of gastroscope insertion in different groups.

Outcome n (%)	Group I (*n* = 15)	Group II (*n* = 15)	Group III (*n* = 15)	Group IV (*n* = 15)	*P*
Failure of gastroscope insertion	6 (40.0)	9 (60.0)	0 (0)*^#^	0 (0)*^#^	<0.001

Group I, Minimal sedation; Group II, Moderate sedation; Group III, Deep sedation; Group IV, Anesthesia.

*P < 0.05 compared with Group I;

^#^*P* < 0.05 compared with Group II. Multiple comparisons were adjusted using the Bonferroni method. Data reporting methods: Qualitative data: n (%). Statistical tests: Chi-square for categorical variables.

#### 3.3.2 Bucking and body movement scores at T2

At T2, bucking and body movement scores showed significant differences among the four groups (*P* < 0.05), with groups III and IV exhibiting significantly lower scores compared to groups I and II (*P* < 0.05) (Table 5).

**TABLE 5:** Bucking and body movement scores during gastroscope insertion (T2) in different groups.

Items [IQR]	Group I (*n* = 15)	Group II (*n* = 15)	Group III (*n* = 15)	Group IV (*n* = 15)	*P*
Bucking scores at T2	1 (1,2)	1 (1,2)	0 (0,0)*^#^	0 (0,0)*^#^	<0.001
Body movement scores at T2	1 (1,3)	2 (1,3)	0 (0,0)*^#^	0 (0,0)*^#^	<0.001

Group I, Minimal sedation; Group II, Moderate sedation; Group III, Deep sedation; Group IV, Anesthesia.

*P < 0.05 compared to Group I;

^#^*P* < 0.05 compared to Group II. Multiple comparisons were adjusted using the Bonferroni method. Data reporting methods: median and interquartile range [M (P25, P75)]. Statistical tests, the Kruskal-Wallis test was used for comparison between groups, and Dunn-Bonferroni correction was performed for pairwise comparison.

### 3.4 Comparison of indicators among participants with successful gastroscope insertion

For participants in whom gastroscope insertion was successfully completed at the start of the procedure, there was a statistically significant difference among the four groups (*P* < 0.05).

The incidence of swallowing, as well as bucking and body movement scores, was significantly lower in groups III and IV compared to groups I and II (*P* < 0.05) (Table 6).

**TABLE 6:** Conditions of participants with successful gastroscope insertion during gastroscopy (T2) in different groups.

Items [n (%)]	Group I (*n* = 9)	Group II (*n* = 6)	Group III (*n* = 15)	Group IV (*n* = 15)	*P*
Swallowing at T2	9 (100.0)	5 (83.3)	2 (13.3)*^#^	0 (0)*^#^	<0.001
Bucking at T2	6 (66.7)	5 (83.3)	2 (13.3)*^#^	1 (6.7)*^#^	<0.001
Body movement at T2	9 (100.0)	5 (83.3)	3 (20.0)*^#^	1 (6.7)*^#^	<0.001

Group I, Minimal sedation; Group II, Moderate sedation; Group III, Deep sedation; Group IV, Anesthesia.

*P < 0.05 compared to Group I;

^#^*P* < 0.05 compared to Group II. Multiple comparisons were adjusted using the Bonferroni method. Data reporting methods: Qualitative data: n (%). Statistical tests: Chi-square for categorical variables.

### 3.5 Change rates of vital signs before and after T2

Among participants with successful gastroscope insertion at the start of the procedure, there were no statistical differences in the change rates of SpO_2_ after T2 (*P* > 0.05). However, there were significant differences in the change rates of HR, SBP, and DBP among the four groups (*P* < 0.05) (Table 7).

**TABLE 7:** Changes in vital signs of participants with successful gastroscope insertion before and after insertion (T2) in different groups.

Characteristics [%, IQR]	Group I (*n* = 9)	Group II (*n* = 6)	Group III (*n* = 15)	Group IV (*n* = 15)	*P*
HR change rates after T2	25.317 (10.738,34.583)	40.635 (9.773,66.689)	0 (0,2.817)*^#^	0 (–3.03,0)*^#^	<0.001
SBP change rates after T2	44.538 (12.27,52.5)	29.444 (5.231,60.448)	0.971 (–5.983,10.101)	–6.25 (–19.355, –1.409)*^#^	<0.001
DBP change rates after T2	15 (8.114,33.691)	11.339 (5.775,36.214)	–1.754 (–7.463,6)*	–11.864 (–22.222, –7.143)*^#^	<0.001
SpO_2_ change rates after T2	0 (–0.5,1.01)	0 (–0.253,0.253)	0 (0,0)	0 (0,0)	0.672

Group I, Minimal sedation; Group II, Moderate sedation; Group III, Deep sedation; Group IV, Anesthesia.

*P < 0.05 compared to Group I;

^#^*P* < 0.05 compared to Group II. Multiple comparisons were adjusted using the Bonferroni method. Data reporting methods: median and interquartile range [M (P25, P75)] HR, Heart Rate; SBP, Systolic Blood Pressure; DBP, Diastolic Blood Pressure; SpO_2_, Peripheral Oxygen Saturation. Statistical tests, the Kruskal-Wallis test was used for comparison between groups, and Dunn-Bonferroni correction was performed for pairwise comparison.

In all participants who experienced significant reductions in SBP and DBP, intravenous administration of norepinephrine (8-10 μg) restored blood pressure to normal levels without causing any subsequent adverse events.

### 3.6 Occurrence of bucking and body movements during gastroscopy (T3)

For participants who achieved successful gastroscope insertion at the beginning of the procedure, there were statistical differences among the four groups (*P* < 0.05).

At T3, the incidence of bucking and body movements was significantly lower in groups II, III, and IV compared to group I (*P* < 0.05) (Table 8).

**TABLE 8:** Conditions of participants with successful gastroscope insertion during gastroscopy (T3) in different groups.

Items [n (%)]	Group I (*n* = 9)	Group II (*n* = 6)	Group III (*n* = 15)	Group IV (*n* = 15)	*P*
Bucking at T3	6 (6.7)	1 (16.7)*	1 (6.7)*	1 (6.7)*	0.002
Body movements at T3	6 (66.7)	1 (16.7)*	2 (13.3)*	0 (0)*	0.001

Group I, Minimal sedation; Group II, Moderate sedation; Group III, Deep sedation; Group IV, Anesthesia.

*P < 0.05 compared with Group I. Multiple comparisons were adjusted using the Bonferroni method. Data reporting methods: Qualitative data: n (%). Statistical tests, Chi-square for categorical variable.

## 4 Discussion

In traditional operating room settings, general anesthesia with muscle relaxation and endotracheal intubation is commonly used for various surgical procedures, where the depth of sedation/anesthesia is typically assumed to be at an anesthetized state. As such, there is no significant issue regarding the selection of an appropriate sedation depth. However, outside the operating room, particularly for various types of non-surgical medical procedures requiring sedation, the choice of an appropriate sedation/anesthesia depth becomes critically important. Comfort and safety are the two core principles of comfortable medical procedures, and different sedation/anesthesia depths correspond to different states of patient consciousness and physical condition. This not only directly impacts the patient’s experience during the procedure but also significantly affects the quality of diagnostic and therapeutic operations, as well as medical safety. For gastrointestinal endoscopy, especially gastroscopy, the psychological stress caused by seeing a long, thick endoscope entering their mouth and twisting inside their body is a concern for patients. However, prior studies have largely lacked targeted research and consensus on the fundamental issue of choosing the appropriate sedation/anesthesia depth for these procedures.

On one hand, many studies on sedation depth are naturally based on local clinical experience, and few studies explain the rationale behind the choice of sedation/anesthesia depth. For example, in procedures such as colonoscopy (15) and ERCP (16), which are more invasive and stimulating, some studies select light or moderate sedation as the target level, while others opt for deep sedation (17). Similarly, Chinese studies on painless gastroscopy have shown varying target sedation depths: Chang et al. (10) chose general anesthesia (MOAA/S score of 0), while Xiajuan et al. (11) selected deep sedation leading to general anesthesia (Ramsay sedation score > 4). However, none of these studies explained the rationale for their choices of sedation/anesthesia depth, relying simply on local clinical practices. Moreover, most studies set a single target sedation/anesthesia depth, and few compare different sedation depths in a single study (18). Even when comparing moderate and deep sedation, studies often fail to include a full spectrum from light sedation to anesthesia.

On the other hand, there are significant differences in the recognition and habitual practices of sedation/anesthesia depth across different countries and regions. A typical example is the study by Kim et al. (19) in Korea, where moderate sedation was implemented using midazolam combined with propofol for painless gastroscopy. This study found that 30-40% of patients exhibited significant body movement during the procedure, and 5-7% required considerable physical restraint to ensure the procedure proceeded smoothly. However, the author described the results as “less than 10% of patients experiencing issues during the procedure,” which, despite being acceptable to the author, does not fully account for the discomfort experienced by those 5-7% of patients requiring significant restraint. Moreover, different countries also have varying practices regarding who administers anesthesia. This has led to definitions differing from anesthesiologist-directed sedation (20), including non-anesthesiologist administration of propofol (21), nurse-administered propofol sedation (22), and endoscopist-directed sedation (23). Some scholars even argue that the involvement of anesthesiologists can increase the use of anesthetic drugs, raise diagnostic and treatment costs, and subject patients to deeper sedation depths (24). Furthermore, for elderly patients, who are often characterized by multiple comorbidities, poor physiological function, and varying drug dosage requirements, there is a lack of targeted design in previous studies regarding sedation/anesthesia depth for this specific population. To summarize, there is a lack of consensus regarding the optimal depth of sedation/anesthesia for specific procedures, particularly in older adults with poor baseline conditions, numerous comorbidities, and high medical safety requirements. To address this gap, the present study established stringent criteria for successful gastroscope insertion and key evaluation indicators, aiming to preliminarily explore the appropriate depth of sedation for painless gastroscopy in older adults.

In this study, a more stringent definition of gastroscope insertion failure was used. According to this criterion, the failure rate of gastroscope insertion was significantly higher in groups I and II compared to groups III and IV. Patients with minimal sedation, who remain fully conscious, can still cooperate with the gastroscopy by following healthcare personnel’s instructions to swallow. In contrast, patients with moderate sedation often exhibit subconscious resistance movements due to impaired consciousness, which can discourage endoscopists from proceeding with scope insertion due to concerns about potential pharyngeal injury. Conversely, patients under deep sedation and anesthesia do not experience these issues, leading to a higher success rate in scope insertion and a smoother start to the gastroscopy procedure in groups III and IV.

At T2, the scores for bucking and body movements were significantly higher in groups I and II compared to groups III and IV. Even among participants with successful gastroscope insertion, the incidence rates of swallowing, bucking, and body movements remained notably higher in groups I and II than in groups III and IV. At T3, group I showed a markedly higher incidence of bucking and body movements compared to the other three groups. In conclusion, minimal and moderate sedation fail to provide a sufficiently stable and quiet environment for endoscopic procedures, resulting in frequent interference from swallowing, bucking, and body movements both during gastroscope insertion and throughout the gastroscopy.

Deep sedation and anesthesia, while providing a more stable environment for endoscopy, significantly impact vital signs compared to minimal and moderate sedation. Our results showed that the change rates of BP were substantially higher in groups III and IV than in groups I and II, both after T1 and T2. The stimulation of gastroscope insertion at T2 did not alleviate hypotension in participants, indicating that deeper sedation and higher doses of propofol adversely affected circulatory stability.

Although there were no significant differences in HR change rates among the four groups at T2, HR was notably lower in groups III and IV at T3 compared to groups I and II. This suggests that HR stability improved with deep sedation and anesthesia, likely due to reduced patient anxiety and fear, despite the decreased perception of injurious stimuli.

Overall, when using deep sedation and anesthesia, proactive management of circulatory stability is crucial. The change rates of SpO_2_ were not significantly different among the groups at either time point, indicating that respiratory function remained stable across all depths of sedation/anesthesia.

The limitations of this study are its single-center nature and that it uses a relatively small sample size, which may affect the generalizability of the findings and introduce potential for false-positive results, such as the observed failure rates of 40 and 60% in groups I and II, respectively, which differ slightly from typical clinical experiences. Additionally, the criteria for sedation failure were set to be relatively stringent to enable precise comparison of different depths of sedation/anesthesia, which may not fully reflect actual clinical practice. Moreover, the study focused on patient conditions at the start of and during gastroscopy, omitting the recovery period from anesthesia and postoperative recovery quality, thus providing an incomplete picture of the overall effects of different sedation depths on patient outcomes.

Collectively, while deep sedation and anesthesia offer a quieter and smoother environment for endoscopy, minimal and moderate sedation have a lesser impact on circulatory stability. For older adults, deep sedation and anesthesia are more appropriate target depths, provided that improved monitoring of vital signs and proactive use of vasoactive drugs are employed to maintain stable circulatory function.

## Data Availability

The raw data supporting the conclusions of this article will be made available by the authors, without undue reservation.
